# New Delhi Metallo-β-Lactamase 1(NDM-1), the Dominant Carbapenemase Detected in Carbapenem-Resistant *Enterobacter cloacae* from Henan Province, China

**DOI:** 10.1371/journal.pone.0135044

**Published:** 2015-08-11

**Authors:** Cailin Liu, Shangshang Qin, Hui Xu, Lijuan Xu, Di Zhao, Xuchun Liu, Shaolei Lang, Xianju Feng, Hong-Min Liu

**Affiliations:** 1 Department of clinical laboratory, The First Affiliated Hospital of Zhengzhou University, Zhengzhou, PR China; 2 Henan province Key Laboratory of Medicine, Zhengzhou, PR China; 3 School of Pharmaceutical Sciences, Zhengzhou University, Zhengzhou, PR China; 4 Department of clinical laboratory, The central hospital of Zhumadian city, Zhumadian, PR China; 5 Department of clinical laboratory, The central hospital of Sanmenxia city, Sanmenxia, PR China; Aligarh Muslim University, INDIA

## Abstract

The emergence of New Delhi metallo-β-lactamase 1 (NDM-1) has become established as a major public health threat and represents a new challenge in the treatment of infectious diseases. In this study, we report a high incidence and endemic spread of NDM-1-producing carbapenem-resistant *Enterobacter cloacae* isolates in Henan province, China. Eight (72.7%) out of eleven non-duplicated carbapenem-resistant *E*. *cloacae* isolates collected between June 2011 and May 2013 were identified as NDM-1 positive. The *bla*_NDM-1_ gene surrounded by an entire IS*Aba*125 element and a bleomycin resistance gene *ble*_MBL_ in these isolates were carried by diverse conjugatable plasmids (IncA/C, IncN, IncHI2 and untypeable) ranging from ~55 to ~360 kb. Molecular epidemiology analysis revealed that three NDM-1-producing *E*. *cloacae* belonged to the same multilocus sequence type (ST), ST120, two of which were classified as extensively drug-resistant (XDR) isolates susceptible only to tigecycline and colistin. The two XDR ST120 *E*. *cloacae* isolates co-harbored *bla*_NDM-1_, *armA* and *fosA3* genes and could transfer resistance to carbapenems, fosfomycin and aminoglycosides simultaneously via a conjugation experiment. Our study demonstrated NDM-1 was the most prevalent metallo-β-lactamase (MBL) among carbapenem-resistant *E*.*cloacae* isolates and identified a potential endemic clone of ST120 in Henan province. These findings highlight the need for enhanced efforts to monitor the further spread of NDM-1 and XDR ST120 *E*. *cloacae* in this region.

## Introduction

*Enterobacter cloacae* (*E*. *cloacae*) is an important nosocomial pathogen causing various infections including urinary tract, skin and soft tissue, lower respiratory tract, wounds, biliary tract, intravenous catheters and central nervous system and intrinsically resistant to ampicillin and narrow-spectrum cephalosporins owing to chromosomal cephalosporinase[1]. Recently, a new antibiotic named Teixobactin was reported to have excellent activity against Gram-positive pathogens without detectable resistance. However, this agent was ineffective against most Gram-negative bacteria containing *Enterobacteriaceae* (*Escherichia coli*: Teixobactin MIC = 25μg/ml; *Klebsiella pneumoniae*: Teixobactin MIC > 25μg/ml)[2]. Due to the increase in multiple drug-resistant Gram-negative bacteria, carbapenems have become the last resort antibiotics in treatment of infections caused by these pathogens including *E*. *cloacae*. The emergence of resistance to carbapenems, mediated by carbapenemases in clinical Enterobacteriaceae such as *E*. *cloacae* isolates represents a serious public health concern worldwide. To date, both metallo-(IMP-8, NDM-1, VIM-1) and non-metallo-(KPC-2) β-lactamases have been reported in carbapenem-resistant *E*. *cloacae*[3–6].

New Delhi metallo-β-lactamase 1 (NDM-1), a metallo-β-lactamase (MBL) capable of hydrolyzing all β-lactams but monobactams, was first identified in a carbapenem-resistant *Klebsiella pneumoniae* strain recovered from a Swedish patient who was hospitalized in India in 2008[7], and mainly detected in carbapenem-resistant *Acinetobacter* spp. in mainland China[8–10]. Only sporadic reports of NDM-1-producing *E*. *cloacae* until the high prevalence and endemic spread of NDM-1-positive *Enterobacteriaceae* was observed in Henan province, China[11]. Thus, the aim of this study is to investigate the current prevalence and molecular characteristics of the NDM-1-producing *E*. *cloacae* in Henan province.

## Materials and Methods

### Bacterial strains and antibiotic susceptibility testing

A total of 112 non-duplicate *E*. *cloacae* clinical isolates were obtained from three hospitals located in the middle [the First Affiliated Hospital of Zhengzhou University (ZZ), n = 69], western [the central hospital of Sanmenxia city (SMX), n = 12], and southern [the central hospital of Zhumadian city (ZMD), n = 31] regions of Henan Province, north-central China from June 2011 to May 2013. Of the 112 isolates tested, 11 isolates (9.8%) (ZZ: n = 7; SMX: n = 1; ZMD: n = 3) were categorized as carbapenem-resistant (Ertapenem, MIC ≥ 2 μg/ml or Imipenem, MIC ≥ 4 μg/ml). All isolates were identified by VITEK2 compact (bioMerieux, France) and 16S rRNA gene sequencing. Antimicrobial susceptibilities for the NDM-1 producing isolates and transconjugants were initially tested using the VITEK2 system and then were followed by measuring the MIC using the broth microdilution method (for imipenem, ertapenem, ciprofloxacin, levofloxacin, gentamicin, amikacin, aztreonam, chloramphenicol and tetracycline), the VITEK2 system (for trimethoprim/sulfamethoxazole, piperacillin/ tazobactam, ceftazidime and cefepime), and the agar dilution method (for fosfomycin), respectively, according to the Clinical Laboratory Standards Institute (CLSI) guidelines(2013). Mueller-Hinton broth (MHB) was used as the test medium in the broth microdilution method, and Mueller-Hinton agar (MHA) containing 25 μg/ml glucose 6-phosphate was used for fosfomycin testing in the agar dilution method. Bacterial suspensions of 0.5 McFarland turbidity for antimicrobial susceptibility testing were prepared by using fresh bacterial colonies taken directly from MHA plates that were incubated at 37°C for 16 to 20 h. Colistin and tigecycline MICs were determined by E-test (AB bioMérieux, France), and results were interpreted as recommended by the European Committee on Antimicrobial Susceptibility Testing (EUCAST 2013). *E*. *coli* ATCC 25922 was used as quality control strain.

### Detection of resistance determinants

All of the carbapenem-resistant *E*. *cloacae* isolates were screened for carbapenemase production by using the modified Hodge test and imipenem-EDTA double-disk synergy test according to the CLSI guidelines. PCR and nucleotide sequencing were employed to screen for the presence of carbapenemases encoding genes[12], extended-spectrum-β-lactamase (ESBL) genes, plasmid-mediated AmpC genes, 16S rRNA methyltransferase genes, and fosfomycin resistance determinants [13–17](Table 1).

**Table 1:** Detection of resistance determinants in the 11 carbapenem-resistant *E*. *cloacae* isolates.

Antimicrobial category	Associated resistance determinants
β-lactams	AmpC genes: *bla*_MOX_, *bla*_CMY_, *bla*_LAT_, *bla*_BIL_, *bla*_DHA_, *bla*_ACC_, *bla*_MIR_, *bla*_ACT_, *bla*_FOX_
	ESBLs genes: *bla*_TEM_, *bla*_SHV_, *bla*_CTX-M_ groups 1, 2, 8, 9 and 26
	Carbapenemase genes: *bla*_IMP,_ *bla*_VIM_, *bla*_KPC_, *bla*_NDM_, *bla*_OXA-1_-like
Aminoglycosides	16S methylase genes: *armA*, *rmtA-E*, and *npmA*
Phosphonic acids (Fosfomycin)	*fosA*, *fosB*, *fosC* and *fosX*,

### Pulsed-field gel electrophoresis (PFGE) and multilocus sequence typing (MLST)

PFGE of XbaI-digested (TaKaRa, Japan) genomic DNA of *bla*_NDM-1_-positive *E*. *cloacae* and reference marker *Salmonella* serotype *Braenderup* strain (H9812) was performed using a contour-clamped homogeneous electric field (CHEF)-Mapper XA PFGE system (Bio-Rad, USA) for 22 h at 6 V/cm and 14°C, with a pulse angle of 120° and pulse times from 5 to 35 s. Comparison of the PFGE patterns was performed with InfoQuest FP software version 4.5 (Bio-Rad Laboratories, USA) using the Dice similarity coefficient. Clusters were defined as DNA patterns sharing >85% similarity. MLST was carried out as described previously[18], the database available at http://pubmlst.org/ecloacae was used for assigning STs.

### Conjugation Experiments

The transfer of carbapenem resistance was tested using a conjugation test (broth mating method), *E*. *coli* J53(sodium azide resistant) was used as the recipient strain. Transconjugants were selected on Mueller-Hinton agar containing sodium azide (100 μg/ml) and imipenem (1μg/ml). The presence of the *bla*_NDM-1_ gene and other resistance determinants according to phenotype in transconjugants were determined by using PCR and sequencing.

### Plasmid analysis and genetic environment of the *bla*_NDM-1_ gene

Plasmid analysis was performed as described previously[19]. Briefly, Genomic DNA was digested with S1 nuclease (TaKaRa, Japan) and separated by PFGE as above, but with a switch time from 2.16 to 63.8 s for 18 h run time. Then, the DNA fragments were transferred to nylon membranes (Millipore, USA), hybridized with digoxigenin-labelled *bla*_NDM-1_-specific probe and detected using a nitroblue tetrazolium-5-bromo-4-chloro -3-indolylphosphate (NBT/BCIP) colour detection kit (Roche Applied Sciences, Germany). The genetic context of the *bla*_NDM-1_ gene was investigated by PCR mapping and subsequent sequencing, the primers were used as described previously[11].

## Results

### Detection of *bla*_NDM-1_ positive isolates

Eight out of the eleven (72.7%) non-duplicate carbapenem-resistant *E*. *cloacae* isolates, were identified as *bla*_NDM-1_ positive, which were obtained from blood (n = 3), urine (n = 2), sputum (n = 2) and wound (n = 1) specimens. Additionally, 2 isolates were IMP-4 positive, and 1 isolate did not contain the carbapenemase genes (*bla*_NDM_, *bla*_KPC_, *bla*_VIM_, *bla*_IMP_, and *bla*_OXA-48-like_) screened in this study. The 8 *bla*_NDM-1_-positive *E*. *cloacae* were obtained from two hospitals in two different cities in Henan Province, including the First Affiliated Hospital of Zhengzhou University (n = 6) and the central hospital of Zhumadian city (n = 2). The clinical data of the 8 isolates were summarized in Table 2. These isolates were collected from 8 individual patients, consisting of 5 male (52.5%) and 3 female (37.5%) with a mean age of 29.7 years, including 2 infants(ECL-2, ECL-36). Of note, 3 patients (37.5%) died of infections.

**Table 2:** Characteristics of *bla*_NDM-1_-positive *E*. *cloacae*.

Isolate	Clinical features				STs^a^	Associated resistance determinants^b^			Plasmid type carrying *bla*_NDM-1_/ Plasmid size (kb)
	Age/Sex	Specimen	Ward	Outcome		β-lactamases	16SrRNA methylase	Others	
ECL-2	7m/female	sputum	Cardial Surgery	discharge	ST177	TEM-1、EBC、CMY-2、CTX-M-1	RmtB	-	Untypeable/70
ECL-4	48y/male	blood	ICU	discharge	ST88	TEM-1、ACT-20、CTX-M-3	-	-	N/65
ECL-27	57y/male	blood	ICU	discharge	ST90	ACT-20、CTX-M-G9	-	-	Untypeable/55
ECL-36	15d/male	sputum	NICU	death	ST41	MIR-2	-	-	A/C/160
ECL-37	37y/male	urine	Urology	discharge	ST120	ACT-20、CTX-M-3	-	-	Untypeable/55
ECL- 62	25y/female	urine	Neurosurgery	death	ST120	ACT-20、CTX-M-15	ArmA	fosA3	HI2/340
ECL- ZMD10	49y/male	wound	Burn unit	discharge	ST120	-	ArmA	fosA3	Untypeable/360
ECL- ZMD12	21y/female	blood	Hematology	death	ST93	-	ArmA	-	A/C/55

^*a*^ ST: Sequence type determined by multilocus sequence typing (MLST)

^*b*^ Resistance markers that are co-transferred with *bla*_NDM-1_by conjugation are underlined. Minus signs indicate negative results.

### Antimicrobial susceptibility testing and detection of resistance genes

All of the *bla*_NDM-1_ carrying isolates were resistant to carbapenems, cephalosporins, monobactams (aztreonam), β-lactam/β-lactamase inhibitor combinations, trimethoprim /sulfamethoxazole, but susceptible to colistin (MICs ≤ 2 μg/ml), and 5 out of 8 isolates (62.5%) exhibited resistance against tigecycline according to the EUCAST breakpoint, with MICs of ≥ 2 μg/ml (Table 3). The modified Hodge test and imipenem-EDTA double-disk synergy test yielded positive results for all isolates. PCR and sequencing results showed most of the *bla*_NDM-1_-carrying *E*. *cloacae* isolates (6/8, 75%) harbored ESBL genes (*bla*_TEM-1_, *bla*_CTX-M-3_, *bla*_CTX-M-9_, *bla*_CTX-M-15_), AmpC genes (*bla*_ACT-20_, *bla*_CMY-2_, *bla*_MIR-2_), or both. Other carbapenemase-encoding genes, including *bla*_KPC_, *bla*_VIM_, *bla*_IMP_, and *bla*_OXA-48-like_, were not detected in any of the *bla*_NDM-1_-positive isolates. Moreover, 4 isolates (50%) harbored 16S methylase genes (*armA* or *rmtB*), exhibited high-level resistance to amikacin (MIC > 256 μg/ml), and 2 isolates (25%) carried a plasmid-mediated fosfomycin resistance gene, *fosA3* (Table 2).

**Table 3:** Antibiotic susceptibilities of *bla*_NDM-1_-positive *E*. *cloacae* and transconjugants (μg/mL).

Isolate no.^a^	Antibiotics ^b^															
	TZP	CAZ	FEP	IPM	ETP	CIP	LEV	GEN	AMK	SXT	ATM	CHL	TET	FOS	TGC	CST
ECL-2	>256	>256	>256	32	>32	1	1	>256	>256	>320	>256	64	>256	32	2	0.5
ECL-4	>256	>256	>256	64	>32	16	>32	8	16	>320	>256	32	128	64	16	0.5
ECL-27	>256	>256	>256	>64	>32	>32	16	64	2	>320	>256	64	>256	16	3	1
ECL-36	>256	>256	>256	32	>32	<0.25	<0.25	32	<2	>320	256	8	4	8	2	1
ECL-37	>256	>256	>256	16	32	16	>32	>256	>256	>320	>256	32	128	64	3	1
**ECL-62**	**>256**	**>256**	**>256**	**64**	**32**	**16**	**32**	**>256**	**>256**	**>320**	**>256**	**256**	**>256**	**>512**	**4**	**1**
**ECL-ZMD10**	**64**	**>256**	**>256**	**8**	**32**	**>32**	**>32**	**>256**	**>256**	**>320**	**256**	**256**	**256**	**128**	**1**	**1**
**ECL-ZMD12**	**>256**	**>256**	**>256**	**8**	**32**	**>32**	**>32**	**>256**	**>256**	**>320**	**>256**	**256**	**256**	**32**	**3**	**2**
*E*. *coli* Transconjugant Strains																
ECL-2-J53	>256	>256	>256	32	32	1	0.5	64	64	>320	128	8	64	16	1	0.5
ECL-4-J53	>256	>256	>256	32	32	16	32	2	8	>320	128	8	32	16	4	0.5
ECL-27-J53	>256	>256	>256	32	>32	16	8	16	2	>320	>256	32	64	8	1.5	1
ECL-36-J53	64	>256	>256	32	>32	<0.25	<0.25	32	<2	>320	<1	8	8	8	1.5	1
ECL-37-J53	>256	>256	>256	16	32	16	32	16	>64	>320	>256	16	128	32	1.5	1
ECL-62-J53	>256	>256	>256	32	16	16	32	64	>64	>320	>256	32	128	>512	2	1
ECL-ZMD10-J53	64	>256	>256	4	16	32	16	>256	>256	>320	128	256	256	128	1	0.5
ECL-ZMD12-J53	>256	>256	>256	8	32	32	32	>256	16	<20	>256	256	256	32	3	1
EC J53	<4	<1	<1	<1	<0.5	<0.25	<0.25	<1	<2	<20	<1	8	2	2	0.25	0.5

^*a*^ ECL, *E*. *cloacae* strains; For the transconjugants, all were *E*. *coli* J53 harboring plasmids from the respective clinical isolates. All of the *bla*_NDM-1_-positive isolates were multidrug-resistant (MDR) strains, the XDR isolates are highlighted in bold type.

^*b*^ Abbreviations used:TZP, piperacillin/tazobactam (0.5/4-256/4); CAZ, ceftazidime (0.03–256); FEP, cefepime (0.015–256); IPM, imipenem(0.06–64); ETP, ertapenem(0.004–32); CIP, ciprofloxacin (0.004–32); LEV, levofloxacin (0.008–32); GEN, gentamicin (0.25–256); AMK, amikacin (0.5–256); ATM, aztreonam (0.06–256); CHL, chloroamphenicol (0.016–256); TET, tetracycline (0.016–256); FOS, fosfomycin (0.25–512); TGC, tigecycline (0.016–256); CST, colistin (0.016–256). The numbers in parentheses indicate the test range (μg/mL) for each agent.

### Molecular epidemiology

**Fig 1:**
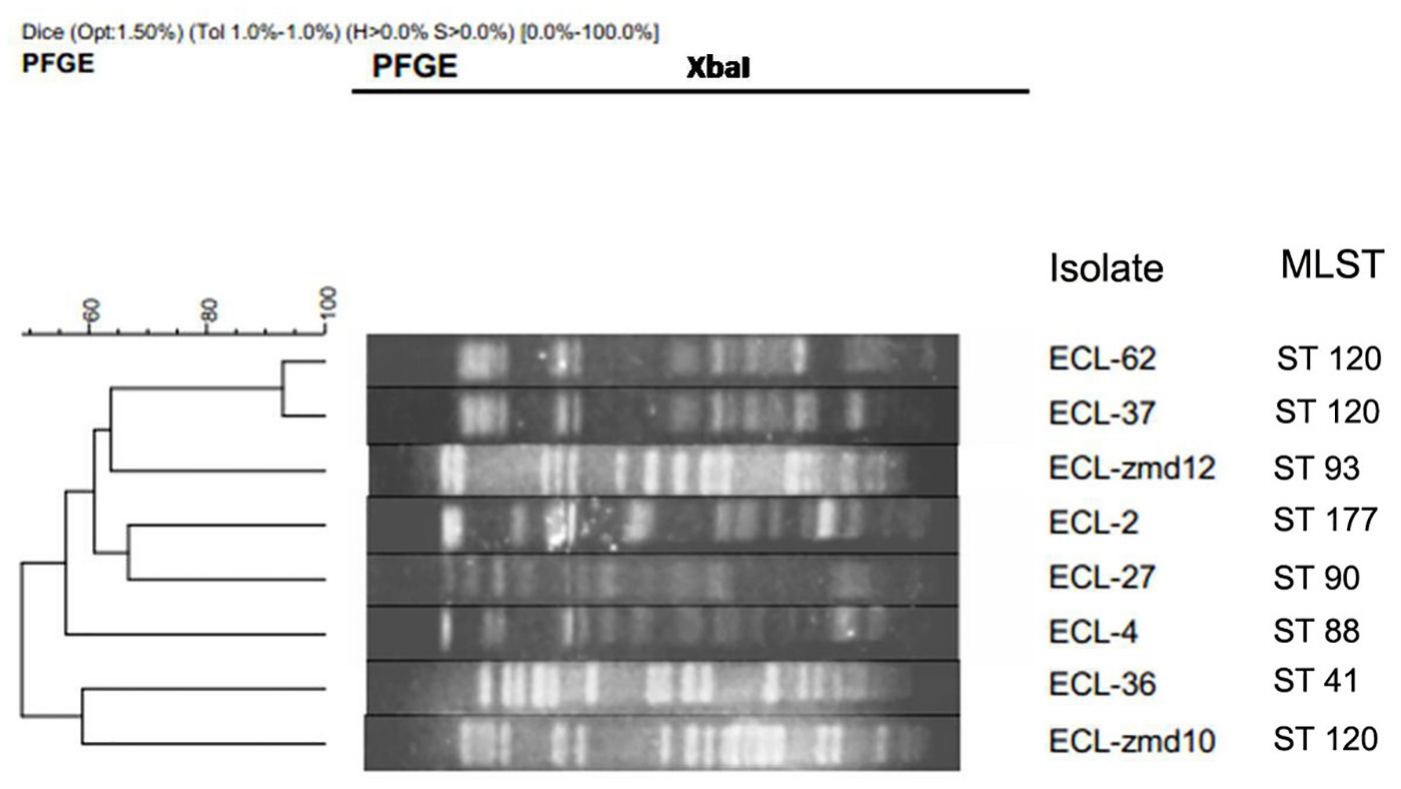
Dendrogram showing pulsed-field gel electrophoresis (PFGE) analysis and multilocus sequence typing (MLST) results for 8 *bla*_NDM-1_-positive *E*. *cloacae* isolates.

Based on a cutoff of 90% genetic similarity, seven PFGE subtypes were identified among the eight isolates. The linkage between PFGE subtype and MLST type was shown in Fig 1. Two isolates obtained from two different wards of the same hospital shared the same PFGE pattern, suggesting they were clonally related, the remaining strains were characterized by unique genotypes. MLST typing revealed 6 STs (ST120[n = 3], ST93[n = 1], ST177[n = 1], ST90[n = 1], ST88[n = 1], and ST41[n = 1]), and 3 isolates belonged to ST120, which were obtained from two different hospitals located in geographically separated areas (the First Affiliated Hospital of Zhengzhou University and the central hospital of Zhumadian city).

### Plasmid analysis and flanking regions of the *bla*_NDM-1_ gene

**Fig 2:**
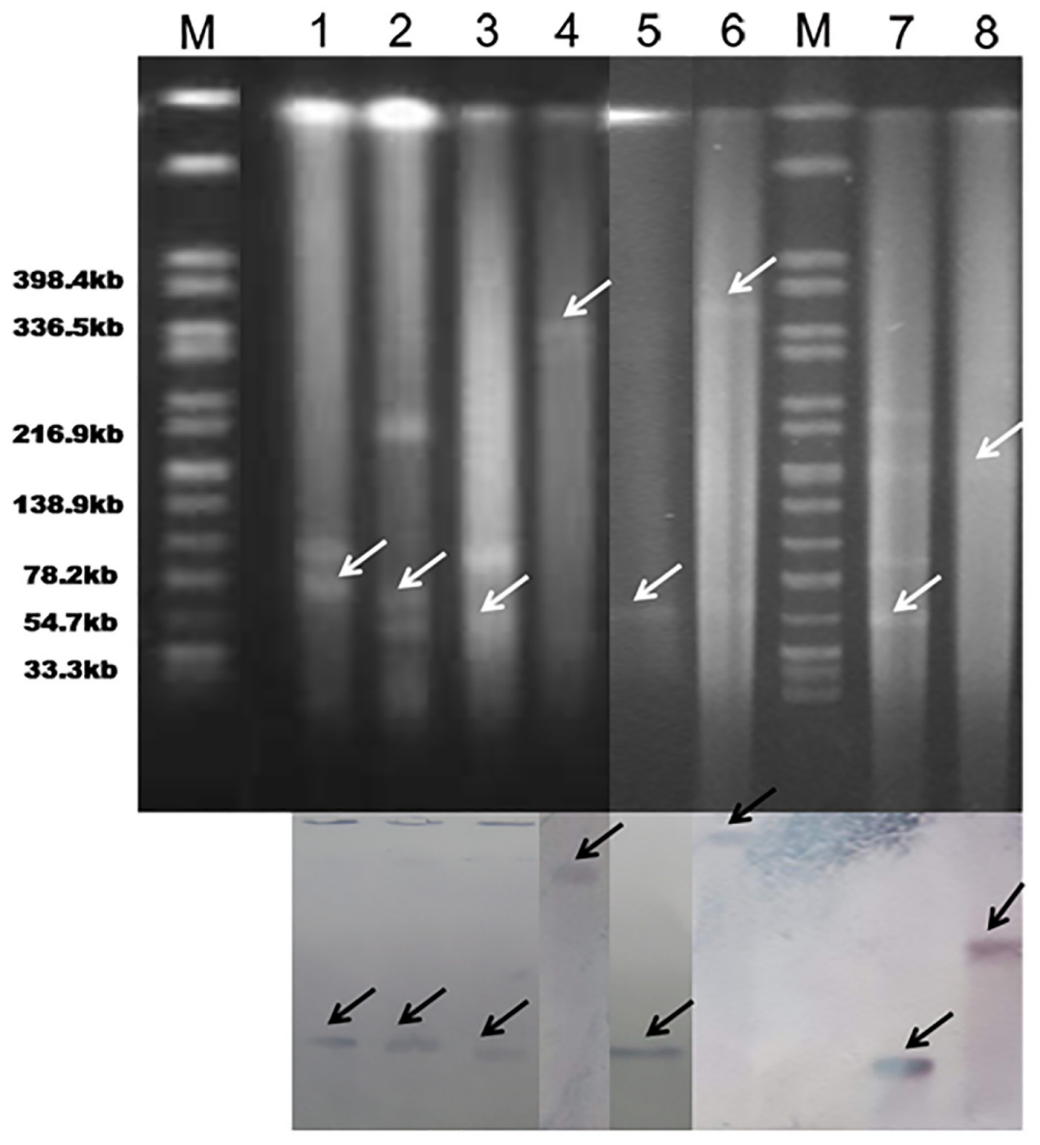
Detection of *bla*_NDM-1_ carrying plasmids by S1 nuclease PFGE and Southern hybridization. Lanes M, marker (*Salmonella* H9812); Lane 1, ECL-2; Lane 2, ECL-4; Lane 3, ECL-37; Lane 4, ECL-62; Lane 5, ECL-27; Lane 6, ECL-ZMD10; Lane 7, ECL-ZMD12; Lane 8, ECL-36.

Conjugation experiments revealed that all of the NDM-1 plasmids were successfully transferred to *E*. *coli* J53, conferring resistance to carbapenems and cephalosporins in transconjugants. In addition, co-transfer of *bla*_NDM-1_ and other resistance determinants (*bla*_TEM-1_, *bla*_CTX-M-3,15/G9_, *bla*_ACT-20_, and *fosA3*) was observed in several isolates (Table 3). The analysis of PFGE profiles of S1 nuclease-digested genomic DNA and Southern blot hybridization showed that *bla*_NDM-1_ was located on diverse plasmids with sizes from~ 55 to~ 360 kb (Fig 2). The NDM-1-encoding plasmids belonged to different plasmid replicon types including IncA/C (n = 2), IncHI2 (n = 1), IncN (n = 1), and untypeable (n = 4) (Table 2 and Fig 2). PCR mapping and sequencing revealed that the entire IS*Aba*125 element was located upstream of *bla*_NDM-1_ and that the bleomycin resistance gene *ble*_MBL_ and truncated *trpF* gene encoding the phosphoribosylanthranilate isomerase were located immediately downstream of the *bla*_NDM-1_ gene in all of the 8 isolates (S1 Fig).

## Discussion

In China, NDM-1 was commonly identified in *Acinetobacter* spp. isolated from clinical, environmental and farm animal samples but only reported sporadically in *Enterobacteriaceae*[8,10,20]. Our recent study demonstrated the prevalence of NDM-1 among carbapenem-resistant *Enterobacteriaceae* (CRE) in Henan province with an incidence of 33.3% and revealed new molecular epidemiological characteristics of CRE in China[11]. As a continued investigation, a pretty high proportion (8/11, 72.7%) of *bla*_NDM-1_ positive strains was identified among carbapenem-resistant *E*. *cloacae* isolates in this study, indicating NDM-1 was the dominant MBL as a mechanism of resistance to carbapenems in *E*. *cloacae* isolates in this region. By contrast, reports from Spain and other southern Europe countries revealed that VIM-1 was the most prevalent MBL among the carbapenem-resistant *E*. *cloacae*[21]. The prevalence rate of carbapenem-resistant *E*. *cloacae* in each hospital (ZZ: 10.1%, 7/69; SMX: 8.3%, 1/12; ZMD: 9.7%, 3/31) in our study was higher than that reported in Spain (5.1%). In addition, a conjugative IncHI2 plasmid of 300 kb plays an important role in dissemination of *bla*_VIM-1_ among different *E*. *cloacae* clones[21], however, NDM-1 plasmids identified in carbapenem-resistant *E*. *cloacae* isolates in this study belonged to multiple replicon types and with various sizes. Observations above demonstrate the importance of the local epidemiological factors in the emergence of specific types of carbapenemases in different regions.

In our study, IS*Aba*125 was located upstream of the *bla*_NDM-1_, while *ble*_MBL_ and a truncated *trpF* gene were located downstream of the *bla*_NDM-1_ in each *E*. *cloacae* isolate, Analysis of the genetic environment of *bla*_NDM-1_ revealed that the region flanking *bla*_NDM-1_ is very similar to some *Acinetobacter* spp. isolated in China. Recent studies highlighted the potential of *Acinetobacter* spp. as a reservoir for the dissemination of NDM-1 towards *Enterobacteriaceae*[22,23]. Given that *bla*_NDM-1_ was mostly detected in *Acinetobacter* spp. in China, we proposed that the acquisition of *bla*_NDM-1_ in *E*. *cloacae* may be originally from *Acinetobacter* spp. under antibiotics selective pressure, and insertion elements may contribute to the spread of *bla*_NDM-1_ among *E*. *cloacae* isolates.

Besides mobile genetic elements mediated *bla*_NDM-1_ transfer, clonal spread is another factor involved in the prevalence of NDM-producing *Enterobacteriaceae* at local and regional level. Outbreaks of NDM-1-producing *Klebsiella pneumoniae* ST147 and ST231 have been reported in Xi’an, China and Ontario, Canada, respectively[24,25]. Our study identified a potential prevalent clone of ST120 among the 8 carbapenem-resistant *E*. *cloacae* isolates in Henan province. However, this ST was different from some widespread *E*. *cloacae* STs (ST66, ST78, ST108 and ST114) that reported in Europe countries, exhibiting expanded-spectrum cephalosporins resistant phenotype [26]. Since limited numbers were obtained, the spread of the ST120 isolates in this region still need to be further monitored. It is noteworthy that two out of the three ST120 isolates (ECL-62 and ECL-ZMD10) were identified as extensively drug-resistant (XDR) bacteria susceptible only to tigecycline and colistin. Moreover, The two XDR ST120 *E*. *cloacae* isolates co-harbored *bla*_NDM-1_, *armA* and *fosA3* genes and could transfer resistance to carbapenems, fosfomycin and aminoglycosides simultaneously by conjugation. Aminoglycosides (gentamycin, amikacin, tobramycin) and fosfomycin were considered as the most common antibiotics for the treatment of infections due to carbapenemase production[27]. The dissemination of *E*. *cloacae* ST120 isolates will seriously limit the future therapeutic options.

In conclusion, our study demonstrated NDM-1 was the most prevalent MBL among carbapenem-resistant *E*. *cloacae* isolates in Henan province, and identified a potential endemic clone of ST120. The emergence of XDR *E*. *cloacae* ST120 isolates is worrying, early detection and surveillance of NDM-1 producing *E*. *cloacae* are urgently needed to prevent their further spread.

## Supporting Information

S1 Fig Genetic environment of the *bla*_NDM-1_ gene in the eight *E*. *cloacae* strains. The boxed arrows indicate the positions and directions of transcription of the genes. The gray-shaded areas represent regions sharing >99% DNA identity.
(TIFF)
