# Correction: New Delhi Metallo-β-Lactamase 1(NDM-1), the Dominant Carbapenemase Detected in Carbapenem-Resistant *Enterobacter cloacae* from Henan Province, China

**DOI:** 10.1371/journal.pone.0140726

**Published:** 2015-10-09

**Authors:** Cailin Liu, Shangshang Qin, Hui Xu, Lijuan Xu, Di Zhao, Xuchun Liu, Shaolei Lang, Xianju Feng, Hong-Min Liu

There is an error in the penultimate sentence of the “Detection of *bla*_NDM-1_ positive isolates” subsection of the Results. The correct sentence is: These isolates were collected from 8 individual patients, consisting of 5 male (62.5%) and 3 female (37.5%) with a mean age of 29.7 years, including 2 infants (ECL-2, ECL-36).

There is an error in Table 2, “Characteristics of *bla*_NDM-1_-positive *E*. *cloacae*.*”* Please see the corrected Table 2 here.

**Table 2:** Characteristics of *bla*_NDM-1_-positive *E*. *cloacae*.

Isolate	Clinical features				STs^*a*^	Associated resistance determinants^*b*^			Plasmid type carrying *bla*_NDM-1_/ Plasmid size (kb)
	Age/Sex	Specimen	Ward	Outcome		β-lactamases	16SrRNA methylase	Others	
ECL-2	7m/female	sputum	Cardial Surgery	discharge	ST177	TEM-1、EBC、CMY-2、CTX-M-1	RmtB	-	Untypeable/70
ECL-4	48y/male	blood	ICU	discharge	ST88	TEM-1、ACT-20、CTX-M-3	-	-	N/65
ECL-27	57y/male	blood	ICU	discharge	ST90	ACT-20、CTX-M-G9	-	-	Untypeable/55
ECL-36	15d/male	sputum	NICU	death	ST41	MIR-2	-	-	A/C/160
ECL-37	37y/male	urine	Urology	discharge	ST120	ACT-20、CTX-M-3	-	-	Untypeable/55
ECL- 62	25y/female	urine	Neurosurgery	death	ST120	ACT-20、CTX-M-15	ArmA	fosA3	HI2/340
ECL- ZMD10	49y/male	wound	Burn unit	discharge	ST120	-	ArmA	fosA3	Untypeable/360
ECL- ZMD12	21y/female	blood	Hematology	death	ST93	-	ArmA	-	A/C/55

^*a*^ ST: Sequence type determined by multilocus sequence typing (MLST)

^*b*^ Resistance markers that are co-transferred with *bla*_NDM-1_by conjugation are underlined. Minus signs indicate negative results.

There is an error in Table 3, “Antibiotic susceptibilities of *bla*_NDM-1_-positive *E*. *cloacae* and transconjugants (μg/mL).” Please see the corrected Table 3 here.

**Table 3:** Antibiotic susceptibilities of *bla*_NDM-1_-positive *E*. *cloacae* and transconjugants (μg/mL).

Isolate no.^*a*^	Antibiotics ^*b*^															
	TZP	CAZ	FEP	IPM	ETP	CIP	LEV	GEN	AMK	SXT	ATM	CHL	TET	FOS	TGC	CST
ECL-2	>256	>256	>256	32	>32	1	1	>256	>256	>320	>256	64	>256	32	2	0.5
ECL-4	>256	>256	>256	64	>32	16	>32	8	16	>320	>256	32	128	64	16	0.5
ECL-27	>256	>256	>256	>64	>32	>32	16	64	2	>320	>256	64	>256	16	3	1
ECL-36	>256	>256	>256	32	>32	<0.25	<0.25	32	<2	>320	256	8	4	8	2	1
ECL-37	>256	>256	>256	16	32	16	>32	>256	>256	>320	>256	32	128	64	3	1
**ECL-62**	**>256**	**>256**	**>256**	**64**	**32**	**16**	**32**	**>256**	**>256**	**>320**	**>256**	**256**	**>256**	**>512**	**4**	**1**
**ECL-ZMD10**	**64**	**>256**	**>256**	**8**	**32**	**>32**	**>32**	**>256**	**>256**	**>320**	**256**	**256**	**256**	**128**	**1**	**1**
**ECL-ZMD12**	**>256**	**>256**	**>256**	**8**	**32**	**>32**	**>32**	**>256**	**>256**	**>320**	**>256**	**256**	**256**	**32**	**3**	**2**
*E*. *coli* Transconjugant Strains																
ECL-2-J53	>256	>256	>256	32	32	1	0.5	64	64	>320	128	8	64	16	1	0.5
ECL-4-J53	>256	>256	>256	32	32	16	32	2	8	>320	128	8	32	16	4	0.5
ECL-27-J53	>256	>256	>256	32	>32	16	8	16	2	>320	>256	32	64	8	1.5	1
ECL-36-J53	64	>256	>256	32	>32	<0.25	<0.25	32	<2	>320	<1	8	8	8	1.5	1
ECL-37-J53	>256	>256	>256	16	32	16	32	16	>64	>320	>256	16	128	32	1.5	1
ECL-62-J53	>256	>256	>256	32	16	16	32	64	>64	>320	>256	32	128	>512	2	1
ECL-ZMD10-J53	64	>256	>256	4	16	32	16	>256	>256	>320	128	256	256	128	1.5	0.5
ECL-ZMD12-J53	>256	>256	>256	8	32	32	32	>256	16	<20	>256	256	256	32	3	1
EC J53	<4	<1	<1	<1	<0.5	<0.25	<0.25	<1	<2	<20	<1	8	2	2	0.25	0.5

^*a*^ ECL, *E*. *cloacae* strains; For the transconjugants, all were *E*. *coli* J53 harboring plasmids from the respective clinical isolates. All of the *bla*_NDM-1_-positive isolates were multidrug-resistant (MDR) strains, the XDR isolates are highlighted in bold type.

^*b*^ Abbreviations used:TZP, piperacillin/tazobactam (0.5/4-256/4); CAZ, ceftazidime (0.03–256); FEP, cefepime (0.015–256); IPM, imipenem(0.06–64); ETP, ertapenem(0.004–32); CIP, ciprofloxacin (0.004–32); LEV, levofloxacin (0.008–32); GEN, gentamicin (0.25–256); AMK, amikacin (0.5–256); ATM, aztreonam (0.06–256); CHL, chloroamphenicol (0.016–256); TET, tetracycline (0.016–256); FOS, fosfomycin (0.25–512); TGC, tigecycline (0.016–256); CST, colistin (0.016–256). The numbers in parentheses indicate the test range (μg/mL) for each agent.
