# Supplementary figures and images for: New Delhi Metallo-β-Lactamase 1(NDM-1), the Dominant Carbapenemase Detected in Carbapenem-Resistant Enterobacter cloacae from Henan Province, China
Source: PLoS One. 2015 Aug 11;10(8):e0135044. doi: 10.1371/journal.pone.0135044 (PMC4532496; doi:10.1371/journal.pone.0135044)

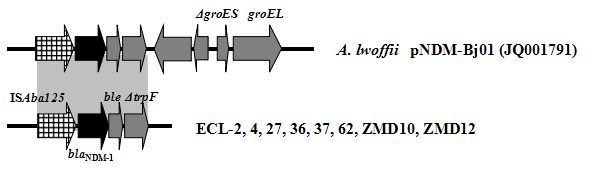

Supplement: S1 Fig — The boxed arrows indicate the positions and directions of transcription of the genes. The gray-shaded areas represent regions sharing >99% DNA identity. (TIFF) [file pone.0135044.s001.tiff]
